# Biodegradable biliary stents in pancreaticoduodenectomy for mitigating biliary complications in high-risk anastomoses

**DOI:** 10.1007/s00464-025-11834-x

**Published:** 2025-06-20

**Authors:** Carolina González-Abós, Claudia Lorenzo, Fabio Ausania

**Affiliations:** 1 HBP and liver transplant Surgery Department, Department of General and Digestive Surgery, Hospital Clínic de Barcelona, IDIBAPS, University of Barcelona, C. Villarroel, 170, 08036 Barcelona, Spain; 2 Gene therapy and cancer, Instituto de Investigaciones Biomédicas August Pi i Sunyer (IDIBAPS), Barcelona, Spain; 3 Universitat de Barcelona, Barcelona, Spain

**Keywords:** Pancreatic surgery, Biliary anastomosis, Biodegradable stents, Biliary fistula

## Abstract

**Background:**

Biliary complications are a significant challenge in pancreaticoduodenectomy (PD), particularly in patients with small bile ducts. This study evaluated the safety and efficacy of biodegradable biliary stents (BS) in reducing biliary complications and improving outcomes in patients undergoing PD.

**Methods:**

This retrospective study included 80 patients with a high risk of biliary fistula undergoing PD at a single high-volume center. Patients were divided into two groups: those who received a biodegradable biliary stent (BS group, *n*=40) and a retrospective cohort of patients who underwent biliary anastomosis without a stent (nBS group, *n*=40). A 1:1 propensity score matching (PSM) was performed based on key clinical and surgical variables to reduce selection bias. The primary outcome was the incidence of biliary fistulas. Cost analysis was also conducted.

**Results:**

After PSM, 30 matched pairs were analyzed. The BS group had significantly shorter operative times for robotic biliary anastomosis (14 [12–18] vs. 22 [20–27] minutes, *p*<0.001). No biliary fistulas occurred in the BS group, compared to 3 cases (10%) in the matched nBS group (*p*=0.039). Comprehensive Complication Index scores were lower in the BS group (21.2 [0–62] vs. 24 [0–88], *p*=0.047), suggesting reduced overall morbidity. One early biliary stricture occurred in the BS group and one late stricture in the nBS group. No deaths occurred in the BS group, while one patient (3.3%) died in the nBS group (*p*=0.317). Cost analysis showed no statistically significant differences but suggested a trend favoring stent use.

**Conclusion:**

Biodegradable biliary stents reduce biliary fistulas and operative time in PD while demonstrating a favorable safety profile. Early complications such as transient jaundice occurred at low rates, the absence of long-term biliary strictures supports their use as a valuable tool in high-risk biliary anastomoses. Multicenter studies with long-term follow-up are necessary to validate these findings and assess cost-effectiveness.

## Background

Pancreaticoduodenectomy (PD) remains a cornerstone surgical procedure for managing malignant and premalignant lesions in the periampullary region. However, it is technically demanding, with postoperative complications occurring in up to 50% of patients. Among these, biliary fistulas (BFs) are particularly challenging, arising in 3% to 8% of cases [1]. These fistulas, characterized by bile leakage from the bilioenteric anastomosis, are associated with significant morbidity, prolonged hospital stays, and increased healthcare costs [2]. Risk factors for BF include a bile duct diameter ≤ 5 mm, excessive dissection, and suboptimal anastomotic technique [3].

Open pancreaticoduodenectomy (OPD) has traditionally been the standard approach for this procedure, offering surgeons direct access to the surgical field [4]. However, OPD is associated with substantial invasiveness, longer recovery times, and higher postoperative morbidity [5, 6]. More recently, robotic pancreaticoduodenectomy (RPD) has emerged as a minimally invasive alternative, leveraging robotic platforms for enhanced visualization, precision, and dexterity in confined surgical spaces [7]. Despite these advancements, both approaches present unique technical challenges, particularly in creating secure biliary anastomoses. While OPD has a well-established learning curve, RPD is associated with a steeper curve, which further increases the risk of complications such as BFs [8].

Efforts to mitigate biliary complications have included the use of T-tubes and non-degradable biliary stents [9]. However, these measures have shown limited efficacy and are frequently associated with complications such as stent migration, obstruction, and infections [9]. Biodegradable biliary stents represent a promising innovation in high-risk biliary anastomosis, offering temporary support for ductal patency during the critical healing phase while degrading naturally to avoid long-term complications [10, 11]. Early studies suggest that their use may reduce the incidence of BFs, facilitate safer and more efficient anastomotic techniques, and standardize anastomotic diameters [10].

While promising, evidence on the efficacy of biodegradable stents remains limited, particularly when comparing their use in OPD versus RPD. Most studies involve small sample sizes and lack of long-term follow-up. This study aims to address these gaps by evaluating the use of biodegradable biliary stents in patients undergoing both OPD and RPD with bile ducts ≤5 mm. By comparing outcomes between patients with and without stents, the study seeks to assess the safety, feasibility, and impact of biodegradable stents on perioperative complications, anastomotic integrity, and overall surgical outcomes across surgical approaches.

## Methods

This retrospective, single-center observational study evaluated consecutive patients who underwent pancreaticoduodenectomy (PD) with high-risk biliary anastomosis (defined as bile duct diameter ≤5 mm) at our institution. Biodegradable biliary stents (BS) were introduced into routine practice in 2022. To form the comparison group, we retrospectively reviewed institutional records and included a control cohort of patients who underwent PD without BS between January 2021 and the time of stent implementation. Inclusion in the control group was based solely on the presence of high-risk biliary anatomy, without selection based on other clinical features. Patient inclusion in the control group was stopped once a numerically matched group to the BS cohort (n = 40) was obtained. Both open PD (OPD) and robotic PD (RPD) cases were included in both groups. To account for potential baseline imbalances, a 1:1 propensity score matching (PSM) analysis was subsequently performed using preoperative and intraoperative variables.

All procedures were performed at a high-volume tertiary care center specializing in hepatobiliopancreatic surgery. Data were extracted from a prospectively maintained institutional database and supplemented by detailed reviews of surgical videos for robotic cases and clinical records.

Patients were eligible for inclusion if they met the following criteria: (1) underwent PD with bilioenteric anastomosis performed either robotically or via open surgery; (2) had a bile duct diameter ≤5 mm on preoperative imaging and confirmed during the operation; and (3) were operated on after the surgeon (FA) had completed at least 20 PD procedures of the respective surgical approach to ensure proficiency. Patients with preoperative biliary stenting, bile duct dilation (>5 mm), or incomplete perioperative data were excluded from the study.

Patients were divided into two groups based on whether a biodegradable biliary stent (BS) was used during the hepaticojejunostomy. In the BS group, the ARCHIMEDES™ Biodegradable Biliary Stent (AMG International GmbH) was utilized. The stent was inserted into the bile duct lumen after completing the posterior wall of the hepaticojejunostomy to maintain ductal patency and facilitate suturing of the anterior wall. The stent degrades completely within six (Fast) or 12 (Medium) weeks, eliminating the need for removal. The nBS group underwent biliary anastomosis without stents.

All robotic bilioenteric anastomoses were performed using a two-layer technique with 4-0 absorbable barbed sutures in a semi-continuous fashion. All open bilioenteric anastomoses were performed using a two-layer technique with 6-0 Polydioxanone Suture (continuous backwall and interrupted anterior wall). In both OPD and RPD cases, biliary anastomosis was consistently performed after completing the pancreaticojejunostomy to minimize confounding variables related to procedural sequence.

The primary outcome was the incidence of clinically relevant biliary fistulas (BFs) within 30 days, classified using the International Study Group of Liver Surgery (ISGLS) criteria. Secondary outcomes included:

- Operative time for biliary anastomosis for RPD only (extracted from video recordings for RPD cases)
- Postoperative morbidity, including biliary fistula, measured by the Comprehensive Complication Index (CCI) [10].
- Rates of reoperation, readmission, and length of hospital stay.
- Long-term biliary complications, including strictures or stent-related issues.

Biliary leaks were diagnosed based on clinical signs (e.g., bilious drainage, jaundice, abdominal pain), biochemical criteria (drain fluid bilirubin concentration >3 times the serum bilirubin level), and confirmed with imaging studies such as CT or MRCP when clinically indicated

Biliary strictures were defined as pathological narrowings of the bile duct demonstrated by CT or MRI scan leading to cholestasis and jaundice. Early biliary stricture occured within the first 90 days postoperatively. In contrast, late biliary stricture resulting in progressive biliary obstruction requiring intervention occurred beyond 90 days after surgery. Biliary complication was defined as the occurrence of either a biliary fistula, a biliary stricture, or both.

The Comprehensive Complication Index (CCI) was calculated based on all postoperative complications occurring within 90 days after surgery, in accordance with established methodology.

Transient postoperative jaundice potentially caused by stent fragmentation was also collected. In our experience, it refers to a temporary elevation in serum bilirubin levels following surgery, which resolves spontaneously or with minimal intervention. This condition may arise from obstruction due to the fragmentation of an indwelling biliary stent, leading to impaired bile flow. Imaging studies or endoscopic evaluation were used to confirm the presence of stent migration or debris-related biliary obstruction, distinguishing this transient phenomenon from more concerning causes of postoperative jaundice.

All patients followed a standardized perioperative protocol tailored to the surgical approach. RPD patients underwent preoperative imaging to assess bile duct anatomy and received robotic-specific intraoperative protocols. Postoperative monitoring included daily blood tests and imaging as clinically indicated. After discharge, follow-up evaluations were conducted at 1 and 6 months, including abdominal imaging (CT or MRI) and liver function tests. Patients were monitored for additional complications at subsequent follow-ups.

Cost analysis in PD patients were estimated for all groups. For this calculation, we analyzed: 1) the costs for each group according to the cost prediction calculator as Novel Cost Assessment Tool for Surgical Procedures described by Staiger et al. (https://www.assessurgery.com/); and 2) the excess expenditure related to biliary complications which also included the hospital stay to the price stipulated per day in our hospital.

Statistical analysis was performed using SPSS version 26.0 (IBM, Armonk, NY, USA). Quantitative variables were expressed as means ± standard deviations (SD) or medians with interquartile ranges (IQR), while categorical variables were presented as frequencies and percentages. Group comparisons (BS vs. nBS) were made using the Mann–Whitney U test for continuous variables and chi-square or Fisher’s exact test for categorical variables. A *p*-value <0.05 was considered statistically significant.

To reduce selection bias and balance baseline covariates between the stent and non-stent groups, a 1:1 propensity score matching (PSM) was performed. Propensity scores were estimated using a logistic regression model based on relevant preoperative and intraoperative variables, including age, sex, BMI, ASA score, surgical approach (open vs. robotic), and indication for surgery. Matching was conducted using nearest-neighbor matching without replacement and a caliper of 0.2. After matching, balance between groups was reassessed and outcomes were re-analyzed in the matched cohort using the same statistical tests.

This study was conducted in accordance with the Declaration of Helsinki and approved by the Institutional Review Board (Approval Code: HCB/2024/0924). Informed consent was obtained from all patients for the use of anonymized clinical data for research purposes.

## Results

**Fig. 1:**
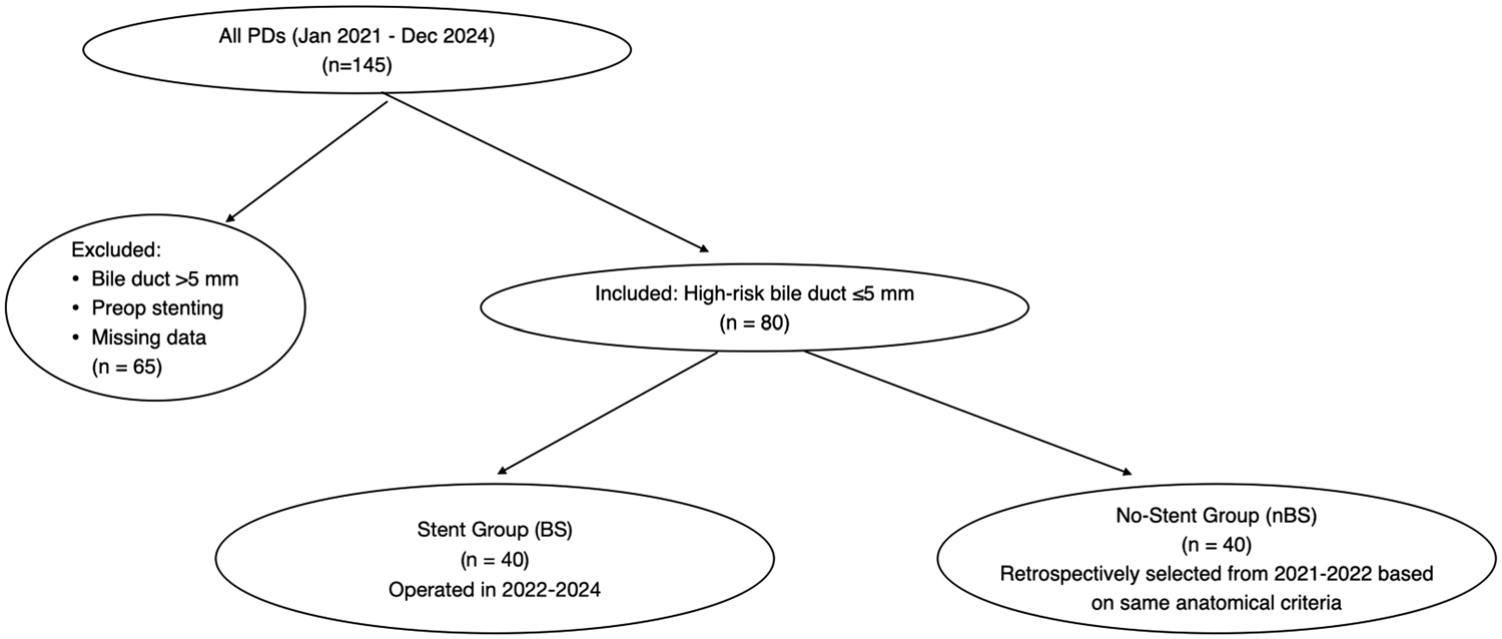
Patient selection flowchart

A total of 80 patients with bile duct diameters ≤5 mm were included in the study: 40 in the biodegradable stent (BS)group and 40 in the no biliary stent (nBS) group. Baseline characteristics were comparable between groups in the unmatched cohort (Table 1), including median age (68 vs. 69 years, *p*=0.826), sex distribution (male: 67.5% vs. 65%, *p*=0.287), body mass index (26 [22–34] vs. 27 [23–35], *p*=0.816), and surgical approach (robotic: 65% vs. 60%, *p*=0.763; open: 35% vs. 40%, *p*=0.763) (Fig. 1).

**Table 1:** Patient baseline characteristics

Characteristic	Biliary Stent (n = 40)	No Biliary Stent (n = 40)	p-Value
Age (years), mean (IQR)	68 (55–73)	69 (54–78)	0.826
Sex ratio (male), n (%)	27 (67.5)	26 (65)	0.287
BMI, mean (IQR)	26 (22–34)	27 (23–35)	0.816
ASA score, n (%)			0.794
II	16 (40)	17 (42.5)	
III	24 (60)	23 (57.5)	
Smoking, n (%)			0.729
Present	4 (10)	3 (7.5)	
Past	4 (10)	7 (17.5)	
Never	32 (80)	30 (75)	
Alcohol consumption, n (%)			0.604
Yes, often	4 (10)	4 (10)	
Yes, occasionally	5 (12.5)	5 (12.5)	
Yes, rarely	24 (60)	22 (55)	
Never	7 (17.5)	9 (22.5)	
Previous abdominal surgeries, n (%)	24 (60)	23 (57.5)	0.954
Indication for surgery, n (%)			
Pancreatic carcinoma	13 (32.5)	15 (37.5)	0.672
IPMN	16 (40)	14 (35)	0.485
Duodenal carcinoma	2 (5)	3 (7.5)	0.382
Neuroendocrine tumor	2 (5)	2 (5)	0.619
Others	7 (17.5)	6 (15)	0.842
Surgical approach, n (%)			0.763
Robotic	26 (65)	24 (60)	
Open	14 (35)	16 (40)	

*BMI*, body mass index; *ASA score*, American Society of Anesthesiologists; *IPMN*, intraductal papillary mucinous neoplasm

To minimize selection bias and improve comparability, a 1:1 propensity score matching (PSM) was performed using key preoperative and intraoperative variables including age, sex, BMI, ASA score, surgical approach, and indication for surgery (Table 2). This yielded two well-balanced groups of 30 patients each (matched BS and matched nBS).

**Table 2:** Perioperative outcomes in unmatched and 1:1 propensity score matched cohorts

Outcome	Unmatched			Matched		
	BS (n=40)	nBS (n=40)	p-Value	BS (n=30)	nBS (n=30)	p-Value
Time for robotic biliary anastomosis (min)	14 (12–18)	23 (21–28)	<0.001	14 (12–18)	22 (20–27)	<0.001
Intraoperative re-do, n (%)	1 (2.5)	2 (5)	0.426	1 (3.3)	1 (3.3)	1.000
Postoperative transient jaundice, n (%)	3 (7.5)	0 (0)	0.092	2 (6.7)	0 (0)	0.150
Early stent migration, n (%)	2 (5)	–	–	2 (6.7)	–	–
Postoperative biliary fistula, n (%)	0 (0)	4 (10)	0.031	0 (0)	3 (10)	0.039
Biliary stricture needing treatment - Early, n (%)	1 (2.5)	0 (0)	–	1 (3.3)	0 (0)	–
Biliary stricture needing treatment - Late, n (%)	0 (0)	1 (2.5)	–	0 (0)	1 (3.3)	–
Biliary complications (fistula + stricture), n of events (%)	1 (2.5%)	4 (10%)	0.031	1 (3.3%)	4 (13.3%)	0.048
Length of hospital stay (days)	11 (5–53)	11 (5–29)	0.757	11 (5–33)	11 (5–25)	0.771
Readmission within 30 days, n (%)	4 (10)	5 (12.5)	0.671	3 (10)	4 (13.3)	0.692
Reoperation within 30 days, n (%)	1 (2.5)	3 (7.5)	0.591	1 (3.3)	2 (6.7)	0.552
Mortality, n (%)	0 (0)	1 (2.5)	0.307	0 (0)	1 (3.3)	0.317
CCI, mean (range)	21.2 (0–62)	23 (0–100)	0.041	21.2 (0–62)	24 (0–88)	0.047
Length of stay in the ICU (days)	1 (1–9)	1 (1–8)	0.433	1 (1–8)	1 (1–8)	0.472
Superficial SSI within 30 days, n (%)	2 (5)	2 (5)	1.000	1 (3.3)	2 (6.7)	0.552
POPF grade B/C, n (%)	7 (17.5)	8 (20)	0.083	6 (20)	7 (23.3)	0.079
PPH grade B/C, n (%)	4 (10)	3 (7.5)	0.741	3 (10)	2 (6.7)	0.682
DGE grade B/C, n (%)	4 (10)	7 (17.5)	0.129	3 (10)	6 (20)	0.085
Chyle leak grade B/C, n (%)	0 (0)	0 (0)	1.000	0 (0)	0 (0)	1.000

Comparison of outcomes in the unmatched and propensity score matched (PSM) cohorts between patients with biodegradable biliary stents (BS) and without stents (nBS)

Perioperative outcomes in both unmatched and matched cohorts are summarized in Table 3. In the unmatched cohort, the median operative time for robotic biliary anastomosis was significantly shorter in the BS group compared to the nBS group (14 [12–18] vs. 23 [21–28] minutes, *p*<0.001), a difference that remained statistically significant after matching (14 [12–18] vs. 22 [20–27] minutes, *p*<0.001).

**Table 3:** Perioperative outcomes

Outcome	Biliary Stent (n = 40)	No Biliary Stent (n = 40)	p-Value
Time for robotic biliary anastomosis (min)	14 (12–18)	23 (21–28)	<0.001
Intraoperative re-do, n (%)	1 (2.5)	2 (5)	0.426
Postoperative transient jaundice, n (%)	3 (7.5)	0 (0)	0.092
Early stent migration, n (%)	2 (5)	-	-
Postoperative biliary fistula, n (%)	0 (0)	4 (10)	0.031
Biliary stricture needing treatment, n (%)			
- Early	1 (2.5)	0 (0)	-
- Late	0 (0)	1 (2.5)	-
Biliary complications (fistula + stricture)	1 (2.5)	4 (10)	0.031
Length of hospital stay (days)	11 (5–53)	11 (5–29)	0.757
Readmission within 30 days, n (%)	4 (10)	5 (12.5)	0.671
Reoperation within 30 days, n (%)	1 (2.5)	3 (7.5)	0.591
Mortality, n (%)	0 (0)	1 (2.5)	0.307
CCI, mean (range)	21.2 (0–62)	23 (0–100)	0.041
Length of stay in the ICU (days)	1 (1–9)	1 (1–8)	0.433
Superficial SSI within 30 days, n (%)	2 (5)	2 (5)	1.000
POPF grade B/C, n (%)	7 (17.5)	8 (20)	0.083
PPH grade B/C, n (%)	4 (10)	3 (7.5)	0.741
DGE grade B/C, n (%)	4 (10)	7 (17.5)	0.129
Chyle leak grade B/C, n (%)	0 (0)	0 (0)	1.000

CCI, comprehensive complication index; POPF, postoperative pancreatic fistula; PPH, postpancreatectomy hemorrhage; DGE, delayed gastric emptying

No biliary fistulas occurred in the BS group, compared to 4 patients (10%) in the unmatched nBS group (*p*=0.031), and 3 patients (10%) in the matched nBS group (*p*=0.039). These findings suggest a consistent reduction in biliary fistulas associated with stent use.

The incidence of biliary strictures was low overall: one patient in the BS group experienced an early stricture requiring intervention, while one late stricture occurred in the nBS group. Importantly, in both cohorts, one patient in the nBS group had both a biliary fistula and a stricture. Therefore, the total number of patients experiencing any biliary complication(fistula and/or stricture) was 1 (2.5%) in the BS group vs. 4 (10%) in the unmatched nBS group (*p*=0.031) and 4 (13.3%)in the matched nBS group (*p*=0.048), demonstrating consistent differences across analyses.

Comprehensive Complication Index (CCI) scores were significantly lower in the BS group in both unmatched (21.2 [0–62] vs. 23 [0–100], *p*=0.041) and matched (21.2 [0–62] vs. 24 [0–88], *p*=0.047) cohorts, reflecting reduced overall morbidity.

The length of hospital stay was similar between groups in both cohorts (median 11 days, *p*>0.7 in both). Readmission and reoperation rates were numerically lower in the BS group in both analyses, though not statistically significant. Mortality occurred in one patient (2.5%) in the nBS group due to sepsis secondary to a POPF, with no deaths recorded in the BS group.

**Fig. 2:**
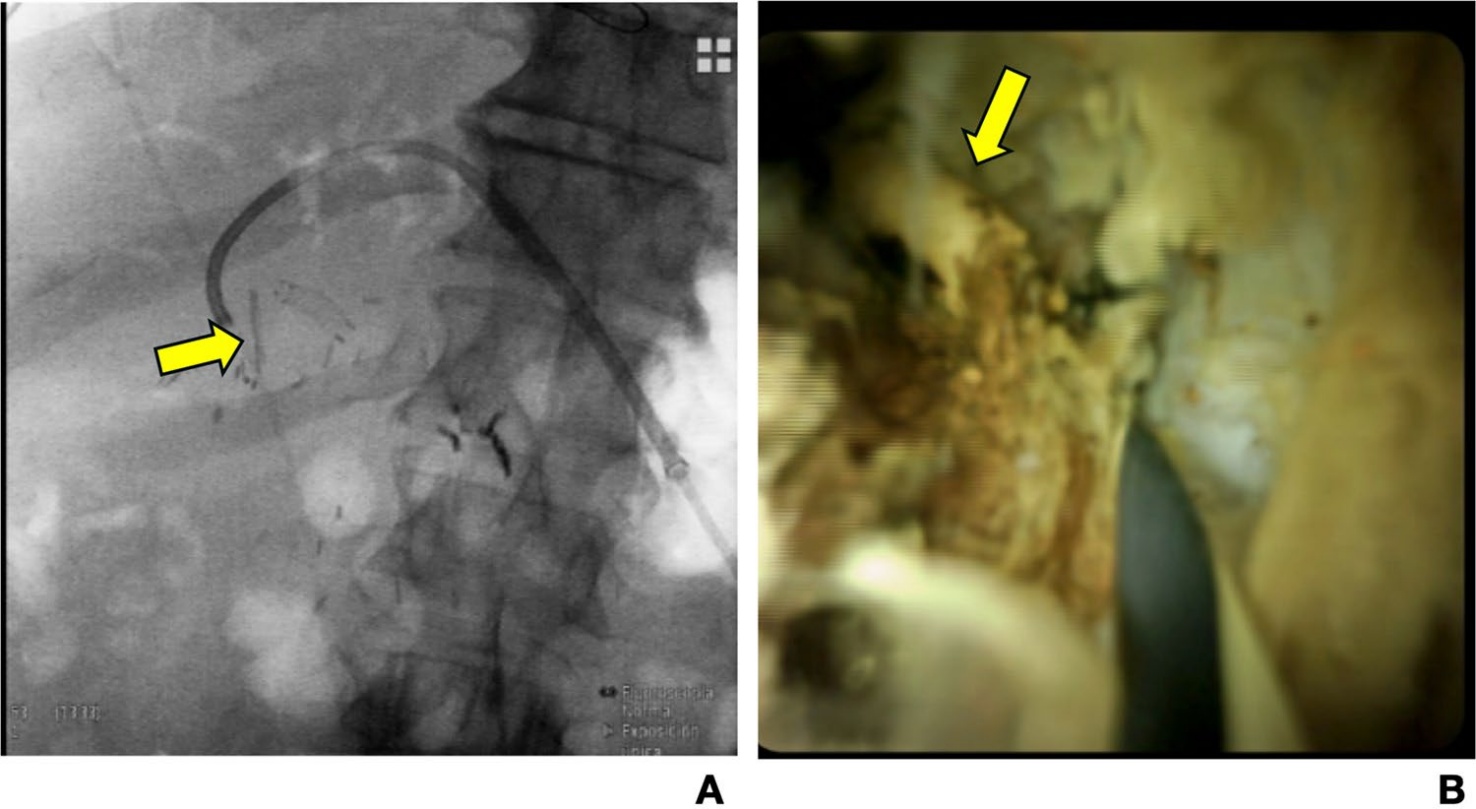
Radiological and endoscopic images showing intrabiliary stent fragmentation. **A**. Radiological images showing the stent fragment, and previous hepatic artery stent. **B**. Endoscopic vision showing the stent fragment wrapped in bile mud

Early stent migration occurred in two patients (5%) in the BS group, with no associated adverse outcomes. One patient with a medium-term degrading stent developed a fragmented obstruction requiring percutaneous drainage and cholangioscopy, as detailed in the complications section. Transient jaundice was noted in 3 patients (7.5%) in the BS group, all resolving spontaneously or with minimal intervention (Fig. 2).

Median follow-up was 13 (1–24) and 20 (6–48) months in BS and noBS groups, respectively (p= < 0.03).

At follow-up, there were no stent-related infections in the BS group.

As summarized in Table 4, There was no statistically significant difference in median total cost between the groups (p = 0.366). However, a clear trend was observed in the cost associated with biliary Complications, tough statistical significance was not achieved due to the low number of patients.

**Table 4:** Cost analysis

	Biliary stent	No biliary stent	p-value
Mean cost/patient, Euros, median (range)	16,812.7 (13,517.4 - 20,107.9)	16,941.3 (13,620.8 - 26,261.8)	0.366
Total biliary complication cost/group, Euros	4200 (1 patient)	11,400 (4 patients)	1

Cost comparison between biliary stent and no biliary stent groups

## Discussion

This study demonstrates that the use of biodegradable biliary stents (BS) in pancreaticoduodenectomy (PD) significantly reduces biliary fistulas and operative time, while maintaining comparable morbidity and mortality rates to conventional approaches without stents. The absence of biliary fistulas in the BS group, along with lower Comprehensive Complication Index (CCI) scores and shorter biliary anastomosis times, underscores the efficacy of BS in optimizing surgical outcomes for patients with small bile ducts [12].

This manuscript represents one the largest single-center investigation to date on the role of biodegradable stents in PD, including a well-balanced cohort of 60 patients. It uniquely addresses both early and late biliary complications, highlighting the safety and utility of BS in maintaining anastomotic patency and minimizing technical challenges during surgery. Unlike prior studies, this research provides a comprehensive analysis of both immediate and delayed biliary outcomes, contributing to a growing body of evidence supporting the clinical adoption of BS in high-risk biliary anastomoses [10].

It is important to highlight several clinically significant findings. "The absence of biliary fistulas in the biodegradable stent (BS) group, compared to a 10% incidence in the matched no stent (nBS) group (*p*=0.039), supports the potential benefit of stent use in reducing biliary fistulas following pancreaticoduodenectomy (PD)." [8, 13]. This contrasts with previous literature reporting biliary fistula rates ranging from 3% to 8% even in high-volume centers with optimized surgical protocols [1]. Also, it has been recently demonstrated that even in experienced hands, the biliary fistula rate following robotic PD can reach 17% in a standard risk cohort of patients [14].

By maintaining ductal patency during healing, biodegradable stents may prevent bile leakage from the anastomotic site, a hypothesis supported by prior smaller-scale studies. However, earlier reports on traditional non-degradable stents have shown mixed results, with complications such as stent migration, infection, or obstruction often negating their benefits [7, 13]. The findings of this study suggest that the biodegradable nature of the stents may eliminate these long-term issues while preserving the advantages of ductal support. However, it is important to note that stent fragmentation causing stricture can be a serious complication, since ERCP may not be possible and a transhepatic approach may be necessary, as in our case, when percutaneous cholangioscopy had to be added to the radiologic approach to obtain clearance of the bilioenteric anastomosis. Therefore, we would recommend to avoid medium-term degrading stents and to limit the stent crossing to the beginning of the bile duct to avoid large fragments proximal to the anastomosis.

Operative time for robotic biliary anastomosis was significantly shorter in the BS group compared to the matched nBS group (14 [12–18] minutes vs. 22 [20–27] minutes, *p*<0.001), suggesting improved technical efficiency during this step. This is consistent with reports from robotic pancreaticoduodenectomy studies, which emphasize the value of adjunctive devices in overcoming the technical challenges posed by small bile ducts [6, 11]. The reduced time likely stems from the stents’ ability to maintain a uniform lumen, facilitating precise suture placement and reducing the risk of posterior wall incorporation. Previous studies have highlighted the learning curve associated with biliary anastomosis, especially in robotic surgery; the findings here suggest that biodegradable stents could serve as a valuable tool in shortening this curve and standardizing outcomes [14].

Comprehensive Complication Index (CCI) scores were lower in the BS group compared to the matched nBS group (21.2 [0–62] vs. 24 [0–88], *p*=0.047), suggesting reduced overall postoperative morbidity.. While this difference is modest, it aligns with existing literature linking reduced biliary complications to lower perioperative morbidity. Early biliary strictures requiring treatment were rare (2.5% in the BS group), matching the lowest rates reported in the literature, and only late strictures were observed in the nBS group. These findings contrast with studies on traditional stents, which report higher rates of stricture formation due to stent-induced fibrosis or chronic inflammation [7, 13].

Early stent migration was observed in 5% of BS patients, consistent with prior reports on biodegradable stents but far below the rates seen with non-degradable stents, which can exceed 20% in some series. These findings underscore the importance of stent design in minimizing adverse events while preserving therapeutic benefits [15]. Moreover, to support the safe adoption of biodegradable stents in clinical practice, we propose the integration of structured postoperative surveillance protocols. These may include early imaging follow-up in symptomatic patients, routine laboratory monitoring, and clearly defined thresholds for investigating abnormal liver function tests. In high-volume or teaching centers, the involvement of a multidisciplinary team—including hepatobiliary surgeons, interventional radiologists, and gastroenterologists—may facilitate timely recognition and intervention in cases of stent-related complications. Additionally, establishing a standardized reporting system for stent-related adverse events across institutions could help gather real-world safety data, inform device refinement, and support the development of best practice guidelines for biodegradable stent use in high-risk biliary anastomoses

The median length of hospital stay was similar between groups (p=0.757), indicating no significant differencereoperation. Notably, no stent-related infections or mortality were observed in the BS group, reinforcing the safety of this approach. Previous studies on non-biodegradable stents have often reported increased infection rates due to biofilm formation, a complication that the biodegradable stents used in this study appear to mitigate [9, 16].

The difference in median follow-up duration between the two groups (13 months in the BS group vs. 20 months in the nBS group) represents a potential limitation of this study. While this discrepancy likely reflects the more recent introduction of biodegradable biliary stents in clinical practice, it could influence the detection of long-term complications, such as late biliary strictures or delayed morbidity. The shorter follow-up period in the BS group may underestimate the true incidence of such events compared to the nBS group, which had a more extended observation period. This discrepancy in follow-up duration inherently limits the ability to capture delayed adverse events, particularly late biliary strictures, which may present months after surgery. As such, our findings regarding long-term safety should be interpreted as preliminary, and ongoing surveillance of this cohort is planned to assess the durability and delayed outcomes associated with stent use.

While our cost analysis did not reach statistical significance, it highlights a clear trend suggesting the potential cost-effectiveness of biliary stenting in minimally invasive distal pancreatectomy. As shown in Table 1, the median total cost for both groups was similar (16,812.7 vs. 16,941.3 euros, p = 0.366), Although the biliary complication cost appeared lower in the stent group, this difference did not reach statistical significance. This trend, despite not achieving statistical significance due to limited sample size, indicates that biliary stenting may be associated with lower complication-related costs. A post-hoc power analysis indicated that, given the observed cost differences, a minimum of 19 patients per group would be required to achieve a statistical power of 80% (with α = 0.05). The inability to reach this threshold in our study suggests that larger cohorts are necessary to definitively confirm these findings. Moreover, our analysis does not account for confounders such as institutional variations, surgeon experience, or postoperative management protocols, all of which could further influence cost outcomes. Future research with larger sample sizes and controlled variables is necessary to validate the economic advantage of biliary stenting and substantiate its role in cost-effective surgical planning.

Apart from the previously discussed limitations of the cost analysis, this study has several additional constraints. The retrospective design introduces potential selection bias, despite comparable baseline characteristics between groups. Conducting this study at a single high-volume center may limit the generalizability of findings, particularly for lower-volume institutions or less experienced surgeons. Additionally, while the sample size of 80 patients allowed for detection of significant differences in biliary fistulas and operative time, it may not fully capture rarer complications or longer-term outcomes. Due to the shorter follow-up duration in the BS group, late complications such as biliary strictures must be interpreted with caution, as some events may not yet have had time to manifest; this represents an inherent limitation of the study design. The lack of detailed cost analysis and patient-reported outcomes further limits the scope of the study. Prospective, randomized multicenter trials with longer follow-up are needed to validate these results.

## Conclusions

The results of this study suggest that biodegradable biliary stents could redefine the standard of care for high-risk biliary anastomoses in PD. By reducing biliary fistulas and simplifying anastomotic techniques, BS may shorten operative times and improve perioperative outcomes, potentially translating to reduced healthcare costs. Additionally, the low incidence of long-term biliary complications highlights their safety and durability. As surgical technologies advance, BS could become integral to robotic and open hepatopancreatic surgery, providing a foundation for further innovations in anastomotic support systems. Future multicenter studies could investigate whether BS use is cost-effective and whether it improves patient-reported outcomes, such as postoperative quality of life.

## Data Availability

The authors confirm that the data supporting the findings of this study are available within the article and its supplementary materials.
